# Earth microbial co-occurrence network reveals interconnection pattern across microbiomes

**DOI:** 10.1186/s40168-020-00857-2

**Published:** 2020-06-04

**Authors:** Bin Ma, Yiling Wang, Shudi Ye, Shan Liu, Erinne Stirling, Jack A. Gilbert, Karoline Faust, Rob Knight, Janet K. Jansson, Cesar Cardona, Lisa Röttjers, Jianming Xu

**Affiliations:** 1 College of Environmental and Resource Sciences, Zhejiang University, Institute of Soil and Water Resources and Environmental Science, Hangzhou, 310058 China; 2 Zhejiang Provincial Key Laboratory of Agricultural Resources and Environment, Hangzhou, 310058 China; 3 Department of Pediatrics and Scripps Institution of Oceanography, University of California San Diego, La Jolla, CA USA; 4 Department of Microbiology and Immunology, Rega Institute, KU Leuven, Campus Gasthuisberg, Leuven, Belgium; 5 Departments of Pediatrics, Computer Science and Engineering, and BioEngineering, University of California San Diego, La Jolla, CA USA; 6 Biological Sciences Division, Earth and Biological Sciences Directorate, Pacific Northwest National Laboratory, Richland, 99352 WA USA; 7 Graduate Program in Biophysical Sciences, The University of Chicago, Chicago, 60637 IL USA

**Keywords:** Co-occurrence patterns, Earth microbiomes, Genelist edges, Network hubs, Negative co-occurrence, Specialist edges, Microbial network topology

## Abstract

**Background:**

Microbial interactions shape the structure and function of microbial communities; microbial co-occurrence networks in specific environments have been widely developed to explore these complex systems, but their interconnection pattern across microbiomes in various environments at the global scale remains unexplored. Here, we have inferred an Earth microbial co-occurrence network from a communal catalog with 23,595 samples and 12,646 exact sequence variants from 14 environments in the Earth Microbiome Project dataset.

**Results:**

This non-random scale-free Earth microbial co-occurrence network consisted of 8 taxonomy distinct modules linked with different environments, which featured environment specific microbial co-occurrence relationships. Different topological features of subnetworks inferred from datasets trimmed into uniform size indicate distinct co-occurrence patterns in the microbiomes of various environments. The high number of specialist edges highlights that environmental specific co-occurrence relationships are essential features across microbiomes. The microbiomes of various environments were clustered into two groups, which were mainly bridged by the microbiomes of plant and animal surface. Acidobacteria Gp2 and Nisaea were identified as hubs in most of subnetworks. Negative edges proportions ranged from 1.9% in the soil subnetwork to 48.9% the non-saline surface subnetwork, suggesting various environments experience distinct intensities of competition or niche differentiation.

Video abstract

**Conclusion:**

This investigation highlights the interconnection patterns across microbiomes in various environments and emphasizes the importance of understanding co-occurrence feature of microbiomes from a network perspective.

## Background

Most microorganisms do not live in isolation; they thrive in communities with large numbers and develop close interactions that generate increased benefits for the group [1, 2]. Microorganisms can establish a range of relationships including mutualism (such as antibiotic resistance conferral), commensalism (such as cross-feeding on compounds produced by other members), synergism (such as syntrophic cooperation), competition (such as niche exclusion), parasitism (such as infecting bacteria), predation (such as ciliates feeding on bacteria), antagosim (such as biocontrol agents), and amensalism (such as inducing a detrimental environment). These ecological interactions are critical evolutionary pressures for natural selection during microbial evolution. This premise is encapsulated by the Red Queen hypothesis, which emphasizes the coevolution of species, wherein established species evolve cooperatively through conditional dependencies [3]. Alternatively, the Black Queen hypothesis, which states that leaky metabolite production in certain species will lead to a reduction in corresponding functions in other species, provides an optional evolutionary possibility for the development of metabolic dependencies [4]. Adaptation in one species may increase selection pressure on another species, giving rise to antagonistic coevolution. Negative interactions (such as competition, parasitism, and predation) drive evolution by selective pressure [5], whereas positive interactions (such as mutualism) drive evolution by enhancing biological fitness [6]. Microbial interactions can partially explain genetic diversity in microbial populations. For example, certain functional genes can be dropped in a microbial genome due to random mutations and selective pressure if those functions are satisfied by other community members, which leads to low and medium gene frequencies, enabling ecological frequency-dependent selective pressure to drive microbial evolution [7]. Meanwhile, microbial interactions could also be reshaped by gaining adaptive genes to extend niche breadth, which alters interaction patterns [8].

Due to the poor mechanistic understanding of microbial community assembly, we found inconsistent prediction performance of microbial community structure in a wide range of fields [9–11]. Solving this unpredictability requires a comprehensive understanding of all aspects of microbiomes, including microbial interaction patterns [2]. Microbial co-occurrence networks are widely applied to explore connections in microbial communities. Nodes and edges in microbial co-occurrence network usually represent microbes and statistically significant associations between nodes, respectively. However, a systematic evaluation of microbial network inference as a tool for interaction prediction has highlighted this tool’s low accuracy and the biological implications of network properties are unclear [12]. Nevertheless, modules in microbial co-occurrence networks may be indicative of ecological processes governing community structure, such as niche filtering and habitat preference [13]. Additionally, microbial co-occurrence network allows to predict hub species and potential species interactions [12]. Global microbial co-occurrence networks can provide a valuable resource for unravelling microbial co-occurrence patterns and their driving mechanisms. Chaffron et al. inferred a global network of co-existing microbes across environments from 298,591 16S rRNA sequences from the Greengenes database and found that phylogenetically close taxa coexisted more frequently [14]. Recent advances of high-throughput sequencing provide an opportunity for predicting microbial co-occurrence patterns from large-scale microbial community studies. For example, Lima-Mendez et al. inferred a global plankton co-occurrence network from the dataset of Tara Oceans, including 313 samples collected from 8 oceanic provinces. This network provides a resource for ocean microbial co-occurrence across several size fractions and depths and demonstrates the value of microbial co-occurrence networks for the formulation of ecological hypotheses such as differences in the role of top-down control across phytoplankton groups. Moreover, this network also helps to determine the role of global trends (generalist edges) and local signals (specialist edges) in driving entire plankton interactome [13]. The Earth Microbiome Project (EMP) is a public database and a framework for crowdsourced sample collection with standardized sequencing and metadata curation [15]. This database provides microbial community resources for cataloging global microbiota at an unprecedented scale for investigating large scale ecological patterns and exploring microbial community assembly theories. Here, we have inferred a global microbial co-occurrence network, describing microbial co-occurrence patterns using a dataset of 23,595 samples encompassing 14 environments from the EMP dataset. We used this network to explore the wired pattern among microbial communities in 14 environments.

## Results

### Earth microbial co-occurrence network

**Fig. 1:**
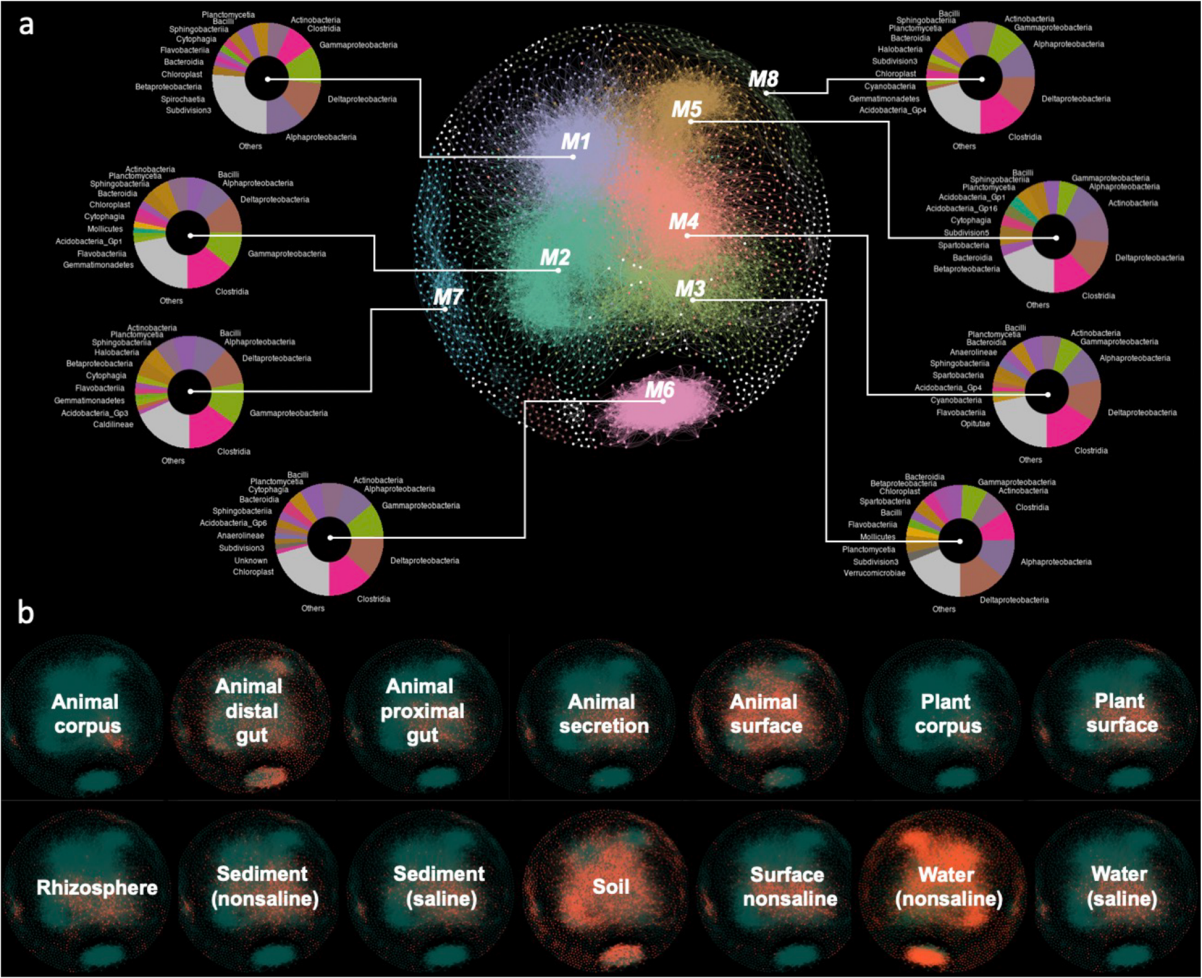
Earth microbial co-occurrence network. **a** Layout and taxonomic profiles of eight domain modules in the Earth microbial co-occurrence network. Modules (M1–M8) are displayed in different colors. **b** The distribution of vertices from 14 environments in the network where orange indicates the vertices from corresponding environments

Fourteen microbial co-occurrence networks representing different environments were constructed, comprising 12,646 exact sequence variants (ESVs). To reduce noise and false-positive predictions, network inclusion was restricted to ESVs present in at least 10% of samples; we also used conservative statistical cut-off values (see “Materials and methods” section). The 14 networks were merged into a single Earth microbial co-occurrence network by overlapping the vertices and edges; the final network consists of 2928 vertices and 54,299 edges after removing unconnected vertices (Fig. 1a). The scale-free property (*R*^2^=0.19, *P*<0.001) and independency between abundance and degree (*R*^2^=− 0.08, *P*=0.07) suggest a non-random co-occurrence pattern in this microbial network (Fig. S1). As ESVs were annotated to their representative microbial taxa, we were able to identify 812 taxa-pairs that were present more than twice in the global microbial co-occurrence network. We validated 432 co-occurrence edges, 15 intra-taxa edges, and 6 competition edges via literature mining (Data file S1). Although this only accounts for 1.5% of edges in the global microbial co-occurrence network, those 812 taxa pairs account for 30% of the edges presented in more than 6 environments.

This global network exhibits a high degree of modularity, but 87.9% of vertices were accounted for by only 8 of the 53 total modules (Fig. S2). Among these eight modules, the first 5 are densely nested into a giant module, while modules 6, 7, and 8 remain isolated from this greater module (Fig. S3). All 8 modules were comprised of different taxonomic profiles and were dominated by Clostridia, Alphaproteobacteria, Deltaproteobacteria, and Gammaproteobacteria (Fig. 1a). Vertices from microbiomes of soils, non-saline waters, animal distal guts, and animal surfaces were present in all 8 modules (Fig. 1b; Fig. S4a), and overrepresented in different modules (Fig. S4b). However, vertices from microbiomes of animal corpus were mostly restricted to and overrepresented than random frequency in M3 (3.1%), while vertices from plant corpus comprised a major portion of and was overrepresented than random frequency in M3 (4.6%) and M4 (2.3%; Fig. S4a-b).

### Phylogeny of co-occurrence network

**Fig. 2:**
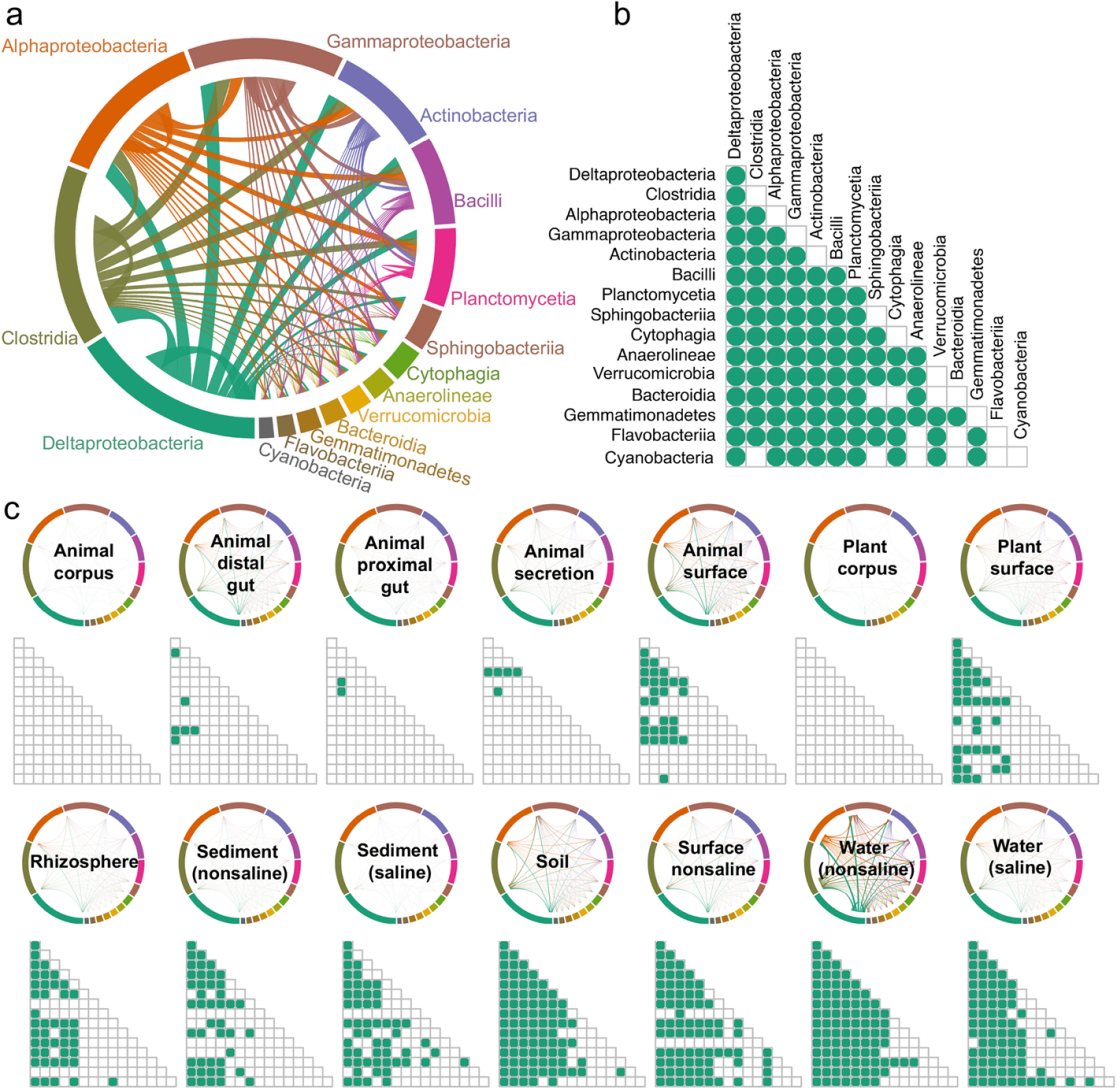
Microbial co-occurrence patterns across dominant taxa. **a** The profiles of co-occurrence links among dominant taxa; note that connections are colored by the most dominant taxon. **b** Overrepresentation of co-occurrence links among taxa. The dot indicates significant overrepresentation (*P*<0.05) between corresponding taxon pairs. **c** The significant overrepresentation of co-occurrence links among taxa in subnetworks for 14 environments

With regard to phylogeny, a non-random edge distribution across taxa was observed, with most co-occurrence relationships derived from Alphaproteobacteria, Clostridia, and Deltaproteobacteria (Fig. 2a) classes. Most of the combinations between dominant classes are overrepresented than random frequency (Fig. 2b). However, only certain combinations between rare classes, such as Flavobacteriia and Gemmatinonadetes, Bacteroidia and Anaeroblineae, and Gemmatinonadetes and Bacteroidia, are overrepresented than random frequency. For within taxa co-occurrence, only co-occurrence within Deltaproteobacteria, Planctomycetia, Anaerolineae, and Acidobacteria Gp2 classes were overrepresented than random frequency. Given that the subnetworks for different environments display different co-occurrence patterns, certain co-occurrence relationships were only overrepresented than random frequency in specific environments (Fig. 2c).

### Topological properties

**Fig. 3:**
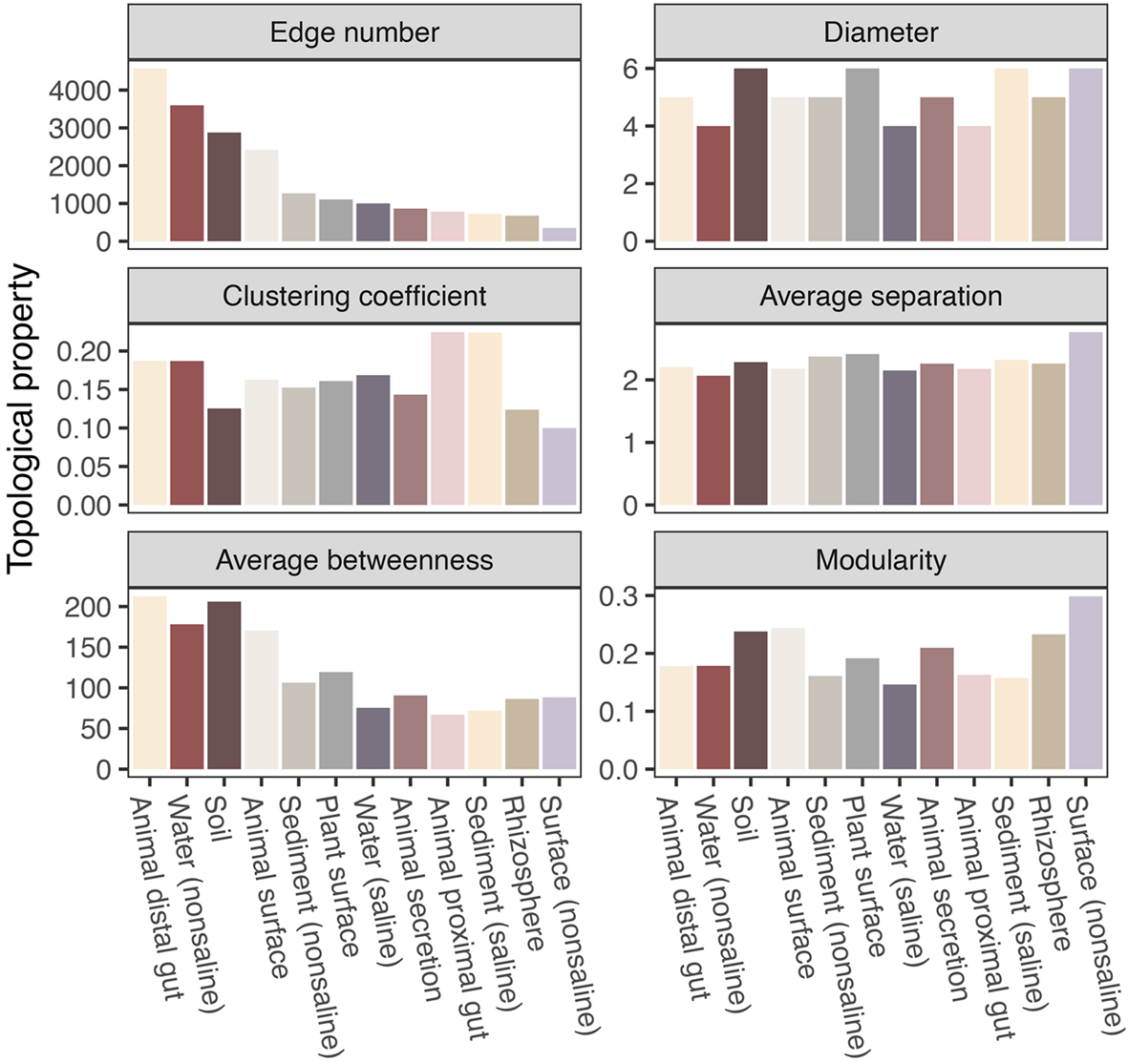
Network topology of subnetworks inferred from trimmed microbiome abundance datasets of 12 environments. The microbiome abundance datasets were trimmed into 400 top-abundant ESVs and random selected 360 samples

To avoid biases introduced by sample number and ESV number, we inferred 12 subnetworks for each environment with datasets trimmed into uniform size (see the “Materials and methods” section). The topological properties were highly variable between the 12 environmental subnetworks (Fig. 3). Although the datasets for 12 environments were trimmed into the same number, the edge numbers of the subnetwork of animal distal gut (4574) was 13 times larger than the subnetwork of non-saline surface (350). The diameter values ranged from 4 to 6 but were not correlated with edge numbers. The clustering coefficient values of subnetworks for animal proximal gut (0.22) and saline sediment (0.22) were higher than of subnetworks for other environments. The average separation (0.30) and modularity (2.7) were the highest for the subnetwork of non-saline surface. Average betweenness centrality values of subnetworks of animal distal gut (212.6) and soil (206.0) were greater than those of other environments.

### Generalist and specialist edges

**Fig. 4:**
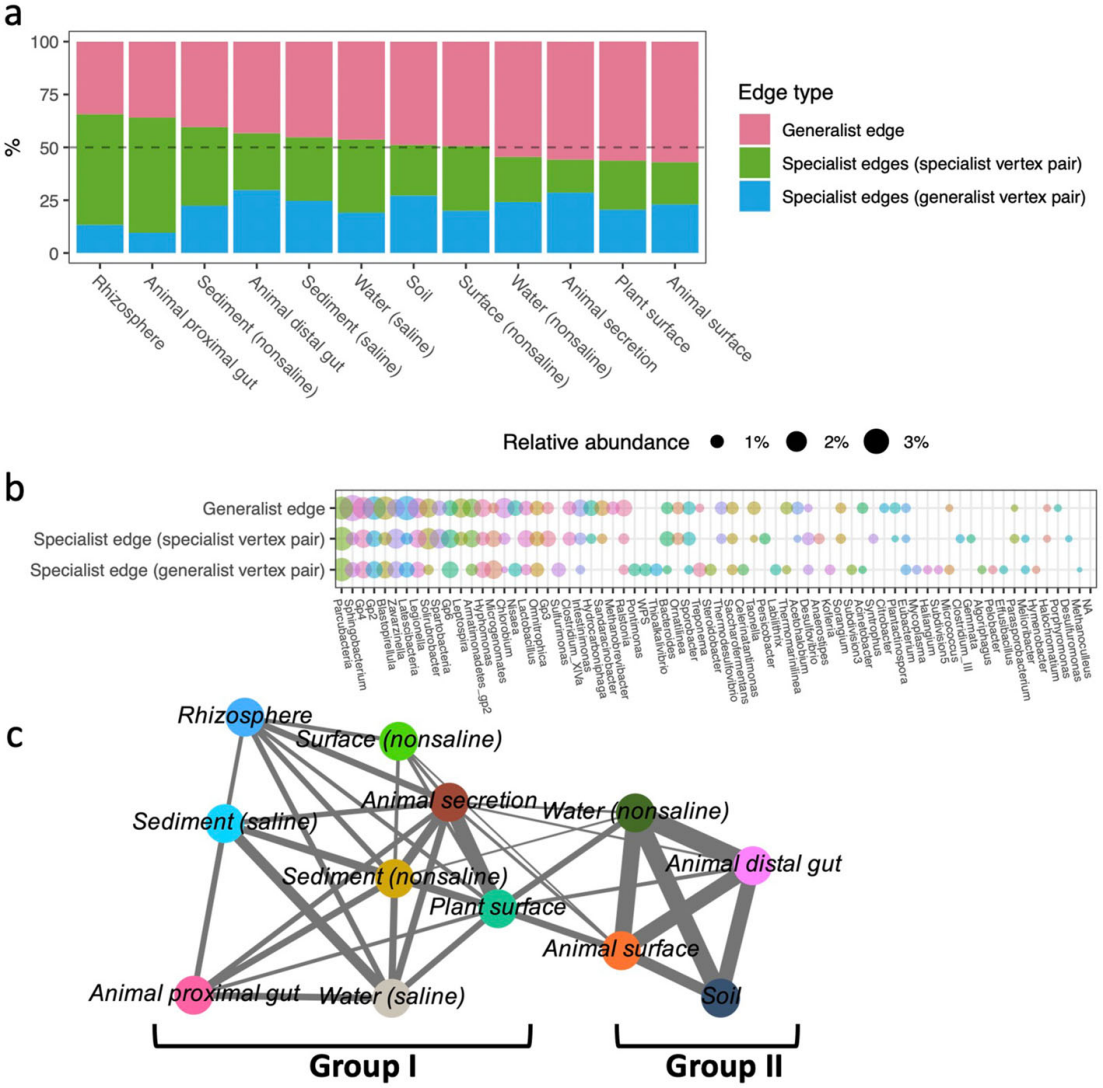
Generalist and specialist edges in subnetworks inferred from trimmed microbiome abundance datasets of 12 environments. **a** Proportions of generalist edges, specialist edges linking specialist vertex pairs, and specialist edges linking generalist vertex pairs in 12 subnetworks. **b** Taxa profiles of vertices associating with generalist edges, specialist edges linking specialist vertex pairs, and specialist edges linking generalist vertex pairs. **c** Interconnection relationships among 12 environments based on similarity of co-occurrence relationships inferred from a Jaccard distance matrix

The proportion of generalist edges, which were present in more than one subnetwork, ranged from 34.3 to 57.0% of the edges in corresponding subnetworks (Fig. 4a). Generalist edges accounted for less than 50% of edges in most subnetworks, except in non-saline water, animal secretion, and the surfaces of plants and animals. The environmental localization of generalist edges was assessed using omission scores (OS, see the “Materials and methods” section). Only 3.4% of generalist edges were identified as local edges (Data file S2).

Specialist edges, which are present in a single subnetwork, could link environment specific vertex pairs present in environment specific subnetworks or link general vertex pairs present in at least two subnetworks. The proportion of specialist edges linking specific vertex pairs accounted for 54.5% of edges in the animal proximal gut subnetwork and 52.4% of edges in the rhizosphere subnetwork, but only accounted for 15.6% of the edges in the animal secretion subnetwork. The proportion of specialist edges linking generalist vertex pairs ranged from 9.6 to 29.8% of edge numbers in corresponding subnetworks; most were greater than 20% except in the animal proximal gut (9.6%), rhizosphere (13.3%) and saline water (19.1%) subnetworks. The proportions of generalist edges were negatively correlated with the proportions of specialist edges linking specific vertex pairs (*ρ*=−0.87, *P*<0.001), but were not correlated with the proportion of specialist edges linking generalist vertex pairs (*ρ*=0.11, *P*<0.72) (Fig. S5). Moreover, the proportions of those three edge types were not related to edge numbers in subnetworks (*P*>0.10) (Fig. S6).

The profiles of the 50 most abundant associated vertices were different for the three edge groups (Fig. 4b). For example, Sphingobacterium was enriched in vertices associated with generalist edges, in which the most abundant edges were Sphingobacterium-Spartobacteria, Sphingobacterium-Legionella, and Sphingobacterium-Solirubrobacter (Data file S2). Microgenomates was enriched in vertices associated with specialist edges linking generalist vertices, in which the most abundant co-occurrence relationships were between Microgenomates and Armatimonates. The taxa profiles of vertices associated with those three edge groups varied with environments (Fig. S7-S9).

Based on edge overlap among the subnetworks inferred from trimmed microbial community data, the 12 environments were clustered into two groups (Fig. 4c). One group consisted of the subnetworks of soil, non-saline water, animal surface, and animal distal gut (group I); the other cluster consisted of the subnetworks for rhizosphere, plant surface, secretion and proximal gut of animal, saline water and sediment, and non-saline sediment and surface (group II). Those two groups were mainly linked through the surface microbiomes of plants and animals.

### Network hubs

**Fig. 5:**
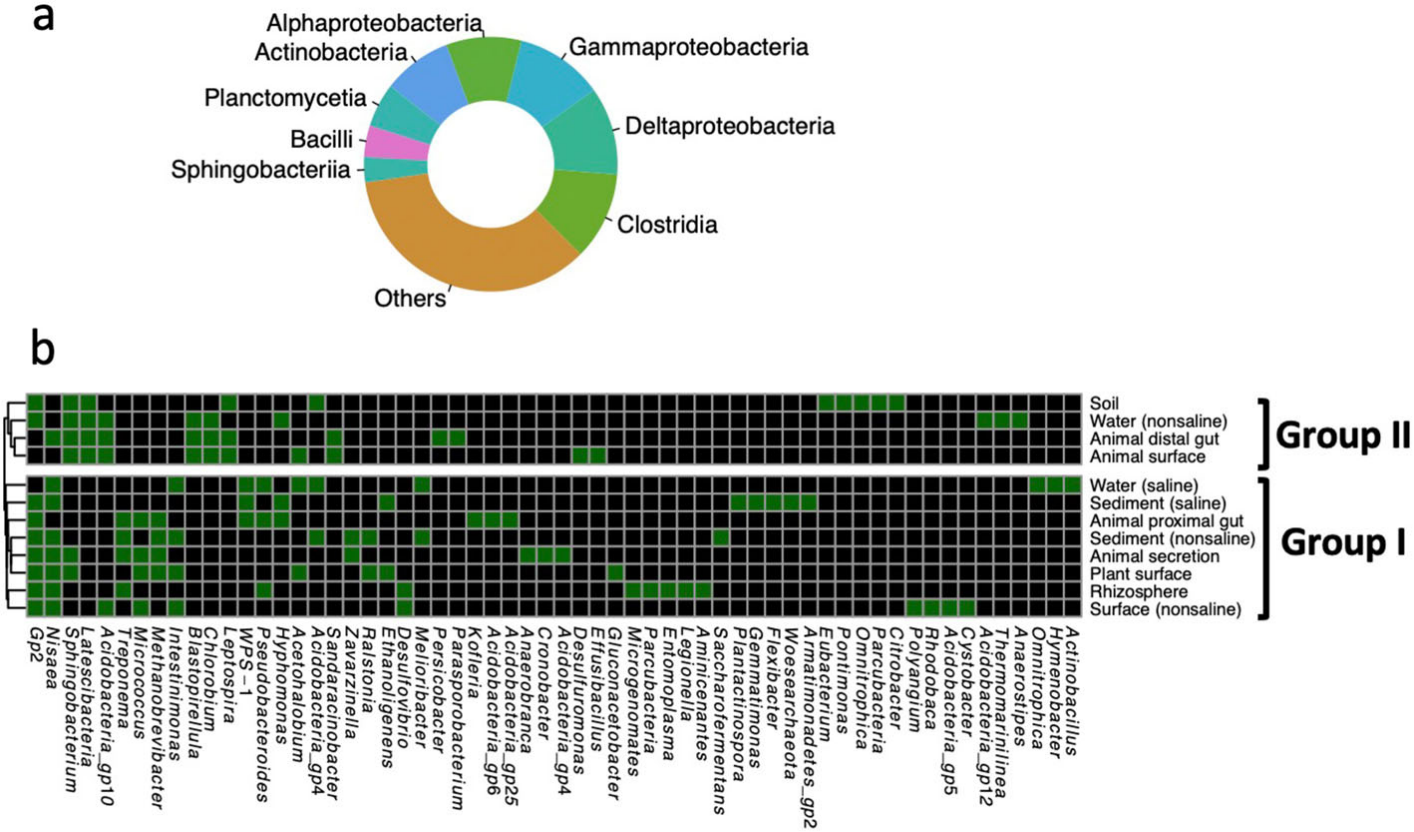
Taxonomic profiles of hub insubnetworks inferred from trimmed microbiome abundance datasets of 12 environments. **a** The class proportion of 120 hubs in 12 subnetworks. **b** The genus profiles of 10 hubs in each subnetwork. The subnetworks for the 12 environments were clustered based on the taxonomic profiles of hubs in the subnetworks

To correct for biases of sample or taxa number, we identified the ten hubs with the highest degree from each subnetwork inferred from 12 trimmed datasets with the same sample and taxa number. A total of 120 hubs belonged to 60 ESVs (Fig. 5a), which were mainly from phyla Clostridia, Deltaproteobacteria, Alphaproteobacteria, Actinobacteria, and Gammaproteobacteria (Fig. 5b). Based on hub presentation, 12 subnetworks were clustered into two groups, which were consist with the two groups clustered based on edge overlap as described above. Acidobacteria Gp2 and Nisaea were identified as hubs in most of subnetworks. Latescibacteria was identified as hubs in all the subnetworks of soil, non-saline water, animal surface, and animal distal gut (group I). Treponema, Micrococcus, and Methanobrevibacter were identified as hubs in four of the subnetworks for rhizosphere, plant surface, secretion and proximal gut of animal, saline water and sediment, and non-saline sediment and surface (group II). Thirty-seven hubs were identified as specialist hubs, which were identified as hubs in only one subnetwork (Fig. 5a), such as in the subnetworks for soil (5), saline sediment (5), and rhizosphere (5).

### Negative co-occurrence links

**Fig. 6:**
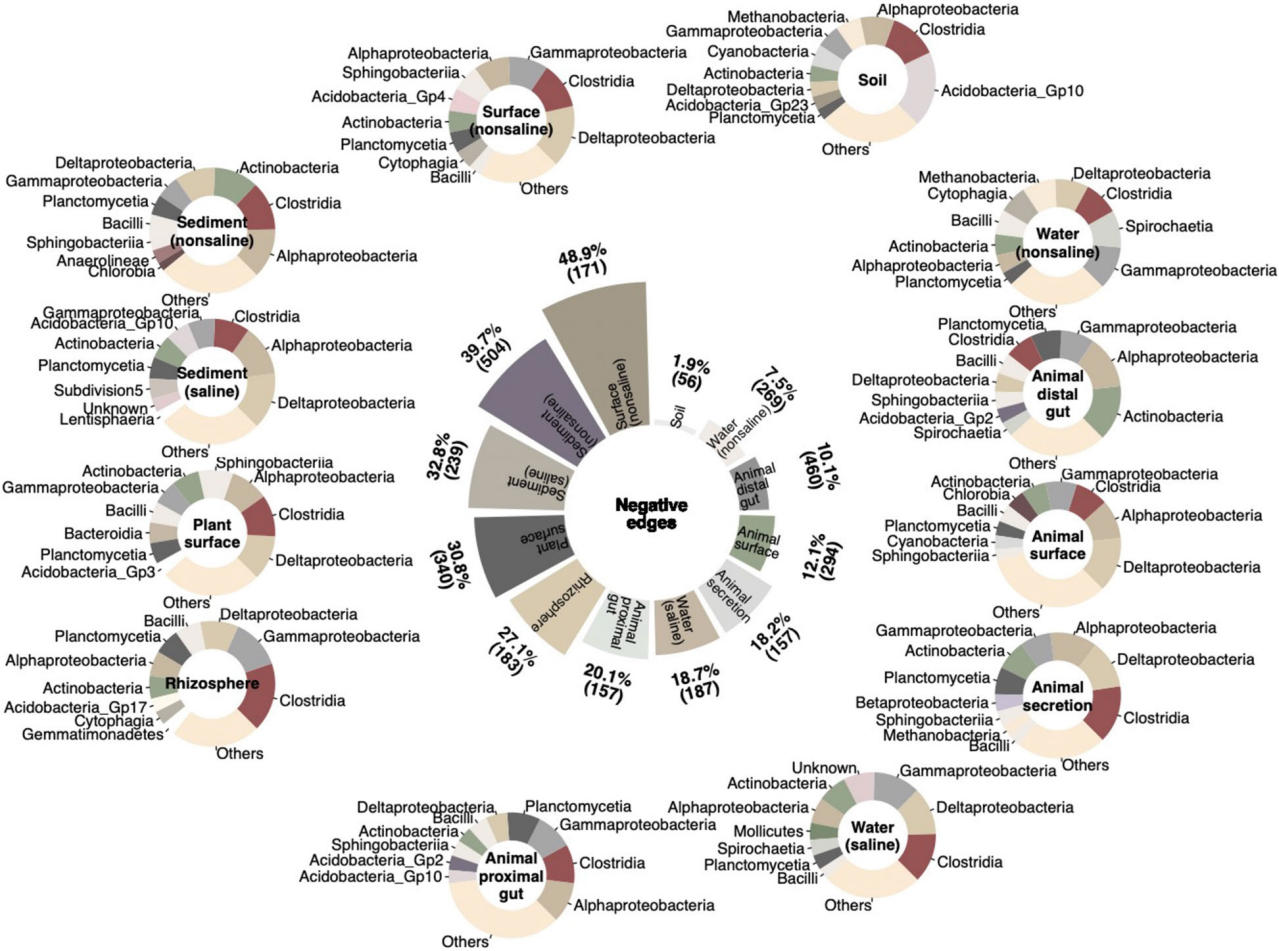
Negative edges in subnetworks inferred from trimmed microbiome abundance datasets of 12 environments. The pie chart in center shows percentage and numbers of negative edges in the 12 subnetworks. The pie charts around the figure’s edge show taxonomic profiles of negative edge associating vertices in the 12 subnetworks

The proportion of negative edges ranged from 1.9 to 48.9% in the 12 subnetworks inferred from trimmed datasets (Fig. 6). Most of subnetworks consisted of more than 10% negative edges, except in subnetworks for soil (1.9%) and non-saline water (7.5%). The proportion of negative edges ranged from 10.1 to 20.1% in the subnetworks for animal associated microbiomes (animal surface, secretion, and distal and proximal gut) and ranged from 27.1 to 30.8% in the subnetworks for plant-associated microbiomes (rhizosphere and plant surface). The proportion of negative edges ranged from 32.8 to 39.7% in the subnetworks for sediments and reached 48.9% in the subnetwork for non-saline surface. Vertices linked with negative edges were dominated by phyla Alphaproteobacteria, Actinobacteria, Clostridia, Deltaproteobacteria, and Gammaproteobacteria, but the taxa profiles of negative edge-linked vertices varied with environments (Fig. 6). A substantial proportion of negative edges were linked with Acidobacteria in the subnetworks of soil, saline sediment, and animal proximal gut, with Spirochaetia in the subnetworks of saline and non-saline water, and with Sphingobacteria in the subnetworks of surface of plant, animal, and non-saline environments. However, most negative edges were environmental specialists at genus level, except for the negative co-occurrence relationships between Spartobacteria and Acidobacteria Gp10, between Legionella and Plantactinospora, and between Acidobacteria Gp6 and Acidobacteria Gp10 (Data file S3).

## Discussion

We generated this global microbial co-occurrence network by taking advantage of the Earth Microbiome Project (EMP) datasets. The global microbial co-occurrence network is scale-free as has been found in other real-world networks such as the world wide web [16], social relationships [17], scientific citations [18], and interactomes of genes [19] and proteins [20]. This scale-free feature implies that a few highly connected hub species coexist with a large number of species that have a small number of links [21] and also implies an ultra-small world network [22], which acknowledges the critical impact of microbial interaction relationships on microbial community assembly processes. Due to the nature of small world networks, impacts affecting one taxon can potentially be delivered to any other member in a microbial community via a few intermediate vertices.

The modularity of this microbial co-occurrence network varied with the environment. Previous studies have shown the existence of environmentally driven modules, such as water depth [23] or soil properties [24]. Moreover, given that modules in microbial co-occurrence networks may represent different niches [2], the present patterns of environments in modules may also indicate a similarity of microbial co-occurrence patterns in different environments. For example, similar module distributions were found for saline water and saline sediment, plant surface and rhizosphere, and the corpus of plants and animals in the current global microbial co-occurrence network.

Different profiles of topological features along the subnetworks of various environments suggest unique microbial co-occurrence patterns in different environments. Large-edge numbers in the animal distal gut subnetwork suggests a high density of interactions in that environment. Higher clustering coefficients in the subnetworks of animal proximal gut and saline sediment might be indicative of cross-feeding relationships, which suggests a richness of degradation pathways, niche filtering, or environmental heterogeneity in such environments. High average separation and modularity in the non-saline surface subnetwork suggests complex ecological processes or environment gradients in non-saline surface. Betweenness centrality measures the centrality of a vertex in a network based on shortest paths. In other words, a vertex with a higher betweenness centrality score would serve as a bridge from one part of a graph to another. The high betweenness centrality indicates that more microbes bridged connections between modules in the subnetworks of animal distal gut and soil.

Given that more than half of the edges were identified as specialist edges in most of the subnetworks, the contribution of specialist edges to co-occurrence patterns was higher than generalist edges. The primary proportion of specialist edges indicates that different environments harbored various specific microbial co-occurrence relationships. Besides specialist edges linking specialist vertices, each environment has its own specialist edges linking taxa pairs that presented in other environments as well. Based on our results, we speculate that microbial co-ocurrences could be an important aspect of describing microbial communities from different environments. Accordingly, microbial co-occurrence patterns provide a new perspective for understanding microbial community assemblages besides taxon composition in microbiomes. Although the previous finding observed major compositional distinction among the microbiomes in soil, non-saline water, animal surface, and animal distal gut, edge overlap among subnetworks indicates a similarity of the microbial co-occurrence patterns among these environments. This cluster of two groups was in line with the subnetwork groups clustered by hub presence as well. The position of plant and animal surface microbiomes might indicate the role of these microbiomes in bridging other microbiomes. However, it is still impossible to validate the existence of inferred edges in different environments at community scale due to the high proportion of uncultured taxa in environmental microbiomes [25] and biases of primers, DNA extraction, and PCR reaction.

Different taxon profiles between generalist edges and specialist edges linking generalist vertices suggest that generalist taxa could display different co-occurrence patterns along different environments. We found that the most abundant generalist edges were all linked with Sphingobacterium, which are ubiquitous in soil [26], water [27], and animal [28] or plant-associating microbiomes [29]. However, ubiquitous existence cannot guarantee formation of generalist edges since substantial specialist edges linked generalist vertex pairs. For example, Microgenomates and Armatimonadetes are co-present in 11 environments, but only formed edges in the animal distal gut subnetwork.

The importance of hub species is intuitive because they are potentially associated with a high number of other species. The high degree of Acidobacteria Gp2 and Nisaea in most of the environments may be explained by their high prevalence and possibly by their generalist lifestyles [30]. Given that Acidobacteria Gp and Nisaea acquired edges with various taxonomic profiles in different environments, those genera may have the potential to synchronize ecological processes over broad ecosystems. Latescibacteria, present in specialist hubs in group I environment subnetworks, is from an uncultured candidate phylum. Its genomic segments recovered from metagenome analyses demonstrate that it is prevalent in a wide range of habitats, but that various Latescibacteria strains prefer specific habitats and have different ecological functions [31]. Latescibacteria could play an important role in the production of cellulosomes in anaerobic habitats, such as in animal guts and sediments, and in polysaccharide degradation in soils [31]. These ecological functions might make Latescibacteria a specialist hub in the subnetworks of group I environments. Treponema, the hub in the subnetwork of animal distal gut, is a characteristic symbiont in human gut microbiomes [32]. Micrococcus, the hub in the subnetworks of plant surface and non-saline surface, plays critical roles in biofilm formation [33]. Methanobrevibacter, the hub in subnetworks of animal distal gut, positively correlated with 20 hydrogen-producing Clostridales in human gut [34]. Moreover, a high proportion of specialist hubs suggests that hubs could represent co-occurrence characteristics in various environments. We note that it is difficult to infer hub nodes correctly [2] and that it is not yet clear whether hub node status also implies a special role in the ecosystem in the sense of a keystone, though initial experiments suggest this is the case [35].

Negative edges may originate from a wide range of co-exclusion mechanisms, including direct competition, toxin production, environmental modification, and differential niche adaptation [36]. Different proportions of negative edges suggest various intensities of competition or niche differentiation in different environments. Low proportions of negative edges in soil subnetworks suggest a prevalence of collaboration or niche sharing in soil, in which heterogeneous microenvironments could reduce direct competition. A large proportion of negative edges in the soil subnetworks linked with Acidobacteria, which are ubiquitous in soil environments but are under-represented in culture studies [37]. The ecological capabilities of Acidobacteria predicted by a metagenomic approach alludes to a competitive life style in soils [37]. High proportions of negative edges in sediments and surface data suggest that competition or subniche differentiation were more prevalent in sediment and surface environments. Compared with the soil, fewer ecological niches exist in sediment and surface environments due to their relatively homogenous microenvironments. The proportion of negative edges in the subnetworks of plant-associated microbiomes was higher than in the subnetworks of animal associated microbiomes, suggesting that competition or niche differentiation was more prevalent in plant-associated microbiomes.

## Conclusions

In summary, the present study provides an overview of global microbial co-occurrence patterns. With this study, we have shown the interconnection pattern across environments in the Earth microbial co-occurrence network. Moreover, we suggest that microbial co-occurrence pattern is a critical aspect of microbial community characteristic that can be used in conjunction with microbial taxon compositional profiles. Given the increasing recognition of the value of communal microbial biodiversity monitoring and the current global advance in sequencing techniques, future sequencing efforts will likely increase the accuracy of the global microbial co-occurrence network presented in this study. Given that most microbial co-occurrence relationships lacked experimental validation, a greater effort is needed to mine uncultured microbial species for validating predicted microbial co-occurrence relationships with co-culture experiments. In addition, the EMP datasets currently focus on bacterial and archaeal communities, but other life forms on Earth (for example plants, animals, and fungi) are also essential in the microbial interactome due to their influences on microbial environments. Future studies filling the gaps for microbial eukaryotes within the EMP framework will untangle global microbial co-occurrence patterns comprehensively.

## Materials and methods

### Abundance table from the EMP

The microbial abundance table used in the present study was the 90-bp Deblur BIOM table from the EMP database [15]. This table was based on the sequence data from the EMP databased after filtering errors and trimming to 90 bp (the length of the shortest sequencing run) using Deblur in Qiime2 [38]. The EMP employed a unified standard workflow for soil collection, metadata curation, DNA extraction, sequencing, and sequence preprocessing, to avoid known issues in combining multiple amplicons across diverse environments on Earth. The abundance table was filtered to keep tag sequences with at least 25 reads total over all samples. We then extracted 14 count matrices for 14 environmental categories at level 3 of the EMP ontology (Table S1) from the 90-bp Deblur BIOM table [15]. We filtered the ESVs with relative abundance less than 0.001% and presenting in less than 10% of samples in corresponding count matrices of environments. All the analyses were done using R 3.6.0 [39].

### Network inference

Microbial taxon-taxon co-occurrence networks were constructed as described by Lima-Mendez et al. [13] by selecting Spearman correlation and Bray-Curtis dissimilarity measures. Briefly, to compute *P* values, we generated permutation and bootstrap distributions (1000 iterations each) by shuffling taxon abundances and resampling from samples with replacements, respectively. The *P* value was then obtained as the probability of the null value under a Gaussian curve fitted to the mean and standard deviation of the bootstrap distribution. Permutations computed for the Spearman correlation included a renormalization step to mitigate compositionality bias. Measure-specific *P*values were merged using Brown’s method [40] and multiple-testing-corrected with the Benjamini-Hochberg method [41]. Finally, edges with an adjusted *P* value above 0.05 and a score below the thresholds determined with random matrix theory method [42] or not supported by both measures after assessment of significance were discarded. For computational efficiency, we computed 14 taxon-taxon networks separately for 14 environmental categories at level 3 of EMP ontology. Network deconvolution was employed for detecting indirect co-occurrences in those networks (*α* =1, *β*=0.9) [43]. The 14 taxon-taxon networks were then merged into a global network.

### Trimmed microbial community dataset

In order to avoid the taxon or sample number biases, we trimmed each community dataset of various environments into the same taxon number and the same sample number. We kept 400 top-abundant ESVs and randomly selected 360 samples in the trimmed microbial community matrices. Due to smaller size than trimmed matrices, the microbiomes in plant and animal corpus were not involved in inferring subnetworks with trimmed dataset.

### Influence of environment on co-occurrence pattern

The impact of environmental categories on the Spearman correlation of each edge in the network was assessed through dividing the absolute omission score (OS) (Spearman correlation without the environmental categories) by the absolute original Spearman score. To account for group size, the OS was computed repeatedly for random, same-sized sample sets. Nonparametric *P* values were calculated as the number of times random OSs were smaller than the sample group OS, divided by the number of random OS (1000 for each taxon pair). Edges were classified as region-specific when the ratio of OS and absolute original score was below 1, and the multiple-testing-corrected *P* values (Benjamini-Hochberg) were below 0.05.

### Overrepresentation analysis

Statistics were done using stats package in R 3.6.0 [39]. Taxon-taxon counts at high taxonomic ranks were assessed for overrepresentation significance using the hypergeometric distribution implemented by *stats*::*phyper*. Mutual exclusion versus co-presence analysis was performed using the binomial distribution implemented by *stats*::*pbinom*, with background probability estimated by the frequency of edges in the network. In all tests, *P* values were adjusted for multiple testing according to Benjamini, Hochberg, and Yekutieli (BY); adjustments were made using the *stats*::*p*.*adjust*.

### Literature-based evaluation of predicted co-occurrence relationships

We counted known species pairs of co-occurrence relationships and filtered the co-occurrence pairs presenting less than three times in the network. Then, we built a list of 812 pairs of species co-occurrence relationships with significant overrepresentation (*P*<0.05). We screened the literature retrieved from Web of Science by querying two species names of a specific co-occurrence relationship and confirm the relationships reported in literature. The protocol to screen the literature was the following: (i) we screened returned literature for direct observed relationship, such as competition or mutualism; (ii) if no direct relationships, we screened returned literature for co-occurrence in the same samples; (iii) if no co-occurrence, we checked if the two species belonged to the same taxon; (iv) otherwise, the edges was labeled as unpublished relationships.

### Topological features

Topological features were estimated with igraph package (v1.4.1) [44] in R 3.6.0 [39]. Edge number was determined using the *ecount* function, and diameter was determined using the *diameter* function. Clustering coefficient was estimated with the *transitivity* function and average separation was estimated with the *mean*_*distance* function. Mean betweenness centrality was calculated using the *centr*_*betw* function. Modularity was estimated with the *modularity* function based on the fast greedy clustering algorithm.

### Generalist and specialist edges

Edges present in only one subnetwork were specialist edges, which were further clustered into two groups: a specialist edge linking a specialist vertex pair or the same linking a generalist vertex pair. A specialist edge linking specialist vertex pair represents the contribution of environmental-specific taxa in specialist edges, while a specialist edge linking generalist vertex pair represents the potential contribution of the environment in enriching specific microbial interactions. The 50 top-abundant ESVs for each edge type were counted for taxon profile comparison. An environment similarity network was inferred with a Jaccard distance matrix based on edge overlap among subnetworks inferred with the trimmed dataset. The spearman correlation between different edge types and between edge number and edge types was calculated using the cor.test function.

### Hub identification

We identified ten hubs at the top-degree from each subnetwork inferred from the 12 trimmed datasets. The taxon profiles of hubs in different subnetworks were identified at genus level with the 90-bp Deblur BIOM table [15].

### Negative edges

We counted the number and percentage of negative edges in the subnetworks inferred from the 12 trimmed datasets. The taxon profiles of negative edges in the subnetworks of various environments were estimated with the class groups of both vertices.

## Supplementary information

Data file S1 Data file S1. Dataset of literature validation records.

Data file S2 Data file S2. Dataset of edge frequency.

Data file S3 Data file S3. Dataset of negative edges.

Supplementary materials Fig. S1. The degree distribution of Earth microbial co-occurrence network. Fig. S2. The relative abundance of vertices in 8 modules of the Earth microbial co-occurrence network. Fig. S3. The co-occurrence across 8 modules of the Earth microbial co-occurrence network. Fig. S4. The distribution of vertices from subnetworks for 14 environmental types among 8 dominant modules. Fig. S5. Relationships between proportions of generalist edge, specialist edge linking generalist vertex pair, and specialist edge linking specialist vertex pair. Fig. S6. Relationship between edge number and generalist edge, specialist edge linking generalist vertex pair, and specialist edge linking specialist vertex pair. Fig. S7. Taxon profiles of generalist edge linked vertices in subnetworks of 12 environments inferred from trimmed datasets. Fig. S8. Taxon profiles of specialist edge linking generalist vertex pair linked vertices in subnetworks of 12 environments inferred from trimmed datasets. Fig. S9. Taxon profiles of specialist edge linking specialist vertex pair linked vertices in subnetworks of 12 environments inferred from trimmed datasets.

## Data Availability

The EMP dataset are available at ftp://ftp.microbio.me/emp/release1. The R script used in this study and network files are available at http://www.github.com/microbma/earthnetwork/.
