# Supplementary material for: Earth microbial co-occurrence network reveals interconnection pattern across microbiomes
Source: Microbiome. 2020 Jun 4;8:82. doi: 10.1186/s40168-020-00857-2 (PMC7273686; doi:10.1186/s40168-020-00857-2)
Supplement: Supplementary file 5 — Fig. S1. The degree distribution of Earth microbial co-occurrence network. Fig. S2. The relative abundance of vertices in 8 modules of the Earth microbial co-occurrence network. Fig. S3. The co-occurrence across 8 modules of the Earth microbial co-occurrence network. Fig. S4. The distribution of vertices from subnetworks for 14 environmental types among 8 dominant modules. Fig. S5. Relationships between proportions of generalist edge, specialist edge linking generalist vertex pair, and specialist edge linking specialist vertex pair. Fig. S6. Relationship between edge number and generalist edge, specialist edge linking generalist vertex pair, and specialist edge linking specialist vertex pair. Fig. S7. Taxon profiles of generalist edge linked vertices in subnetworks of 12 environments inferred from trimmed datasets. Fig. S8. Taxon profiles of specialist edge linking generalist vertex pair linked vertices in subnetworks of 12 environments inferred from trimmed datasets. Fig. S9. Taxon profiles of specialist edge linking specialist vertex pair linked vertices in subnetworks of 12 environments inferred from trimmed datasets. [file 40168_2020_857_MOESM4_ESM.pdf]

Supporting Materials

Earth microbial co-occurrence network reveals interconnection pattern across microbiomes

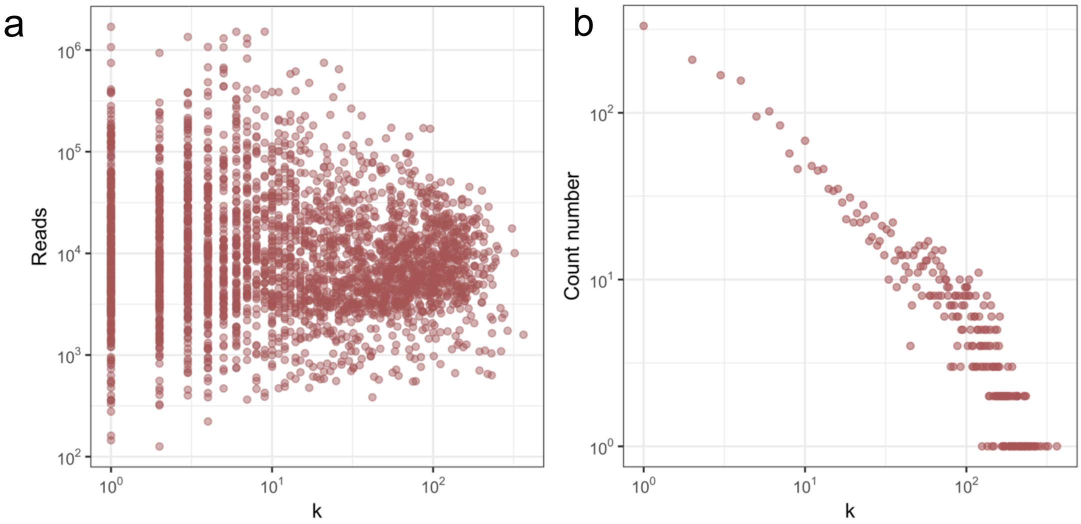

Figure S1. The degree distribution of Earth microbial co-occurrence network. (a) Relationship between degree and the read counts of corresponding ESVs. (b)The scale free distribution of degree of nodes.

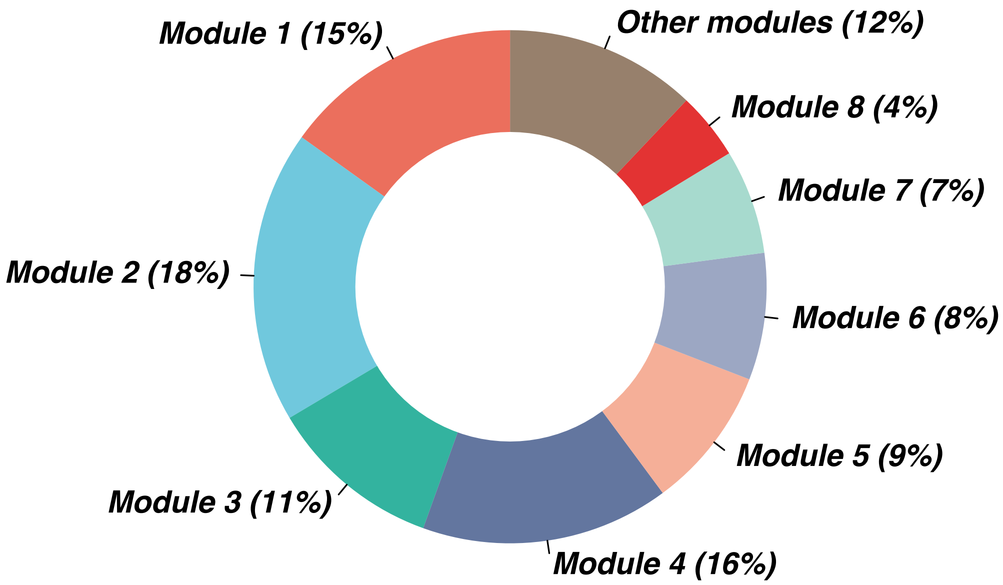

Figure S2. The relative abundance of vertices in 8 modules of the Earth microbial co-occurrence network.

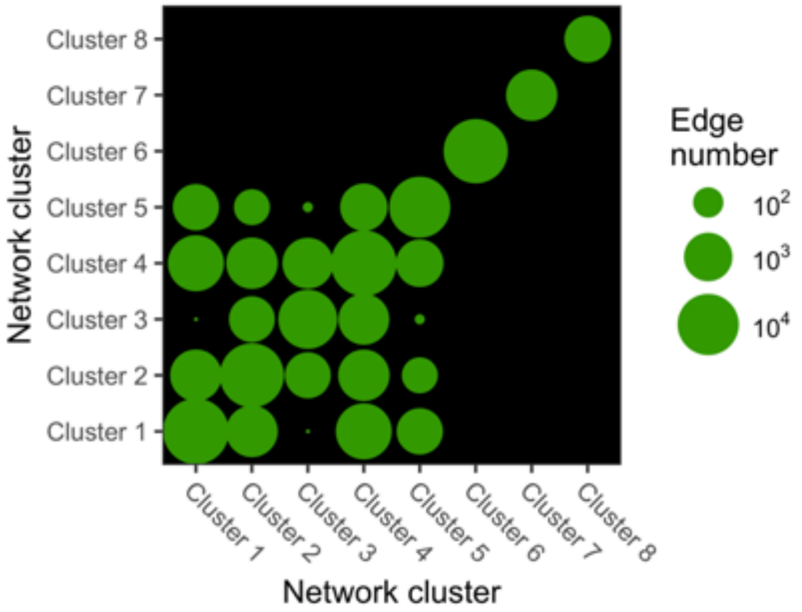

Figure S3. The linkage across 8 modules of the Earth microbial co-occurrence network.

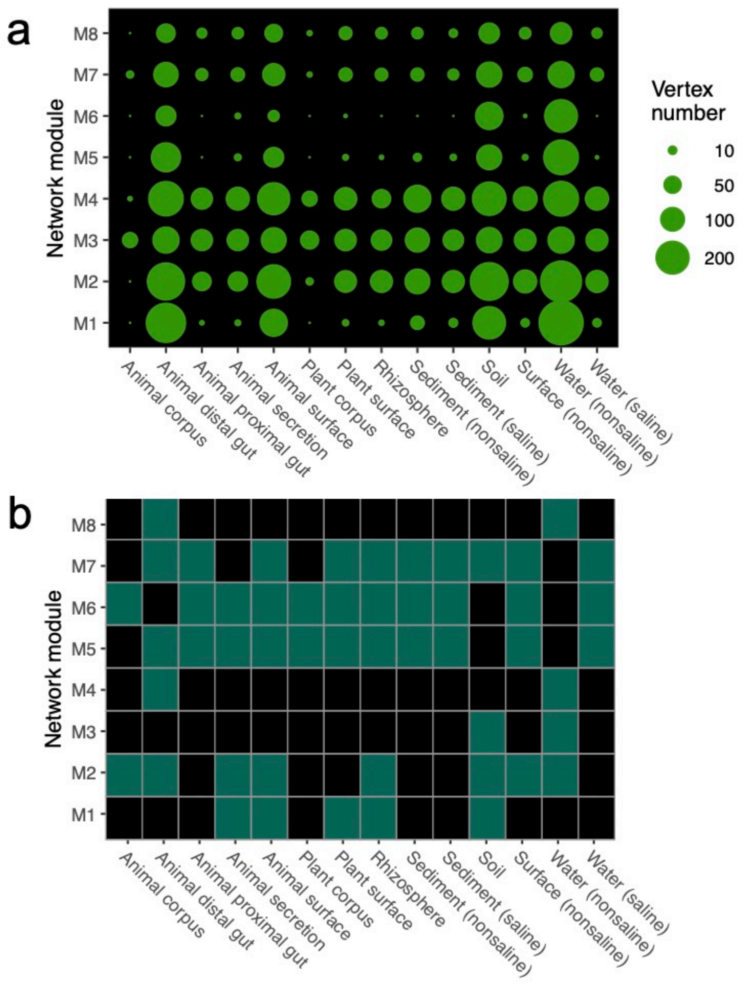

Figure S4. The distribution of vertices from subnetworks for 14 environmental types among 8 dominant modules. (a) Abundance profiles of vertex from 14 environmental type in 8 modules. (b) Overrepresentation of environmental types in 8 modules.

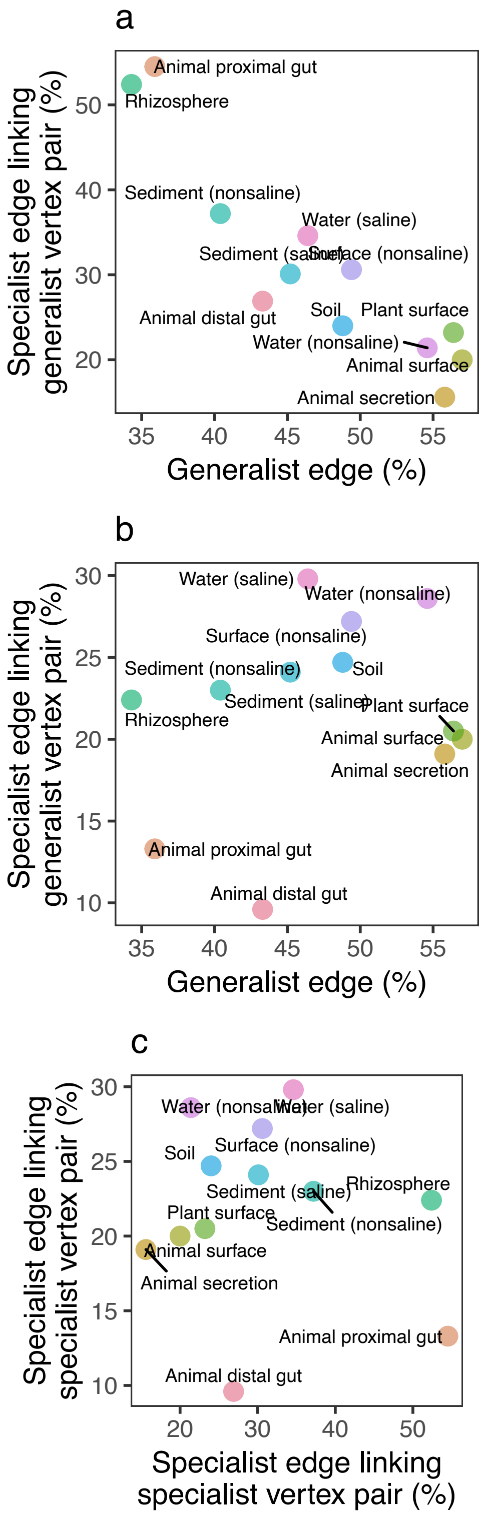

Figure S5. Relationships between proportions of (a) generalist edge and specialist edge linking generalist vertex pair, (b) generalist edge and specialist edge linking specialist vertex pair, and (c) specialist edge linking generalist vertex pair and specialist vertex pair.

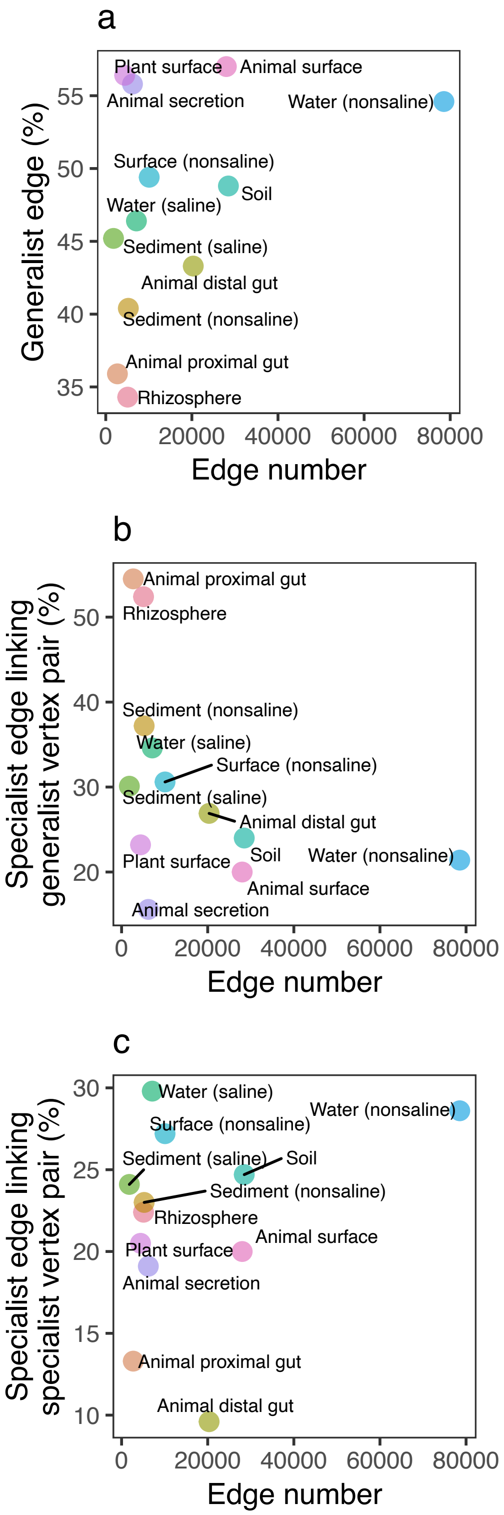

Figure S6. Relationship between edge number and (a) the proportion of generalist edge, (b) specialist edge linking generalist vertex pair, and (c) specialist edge linking specialist vertex pair.

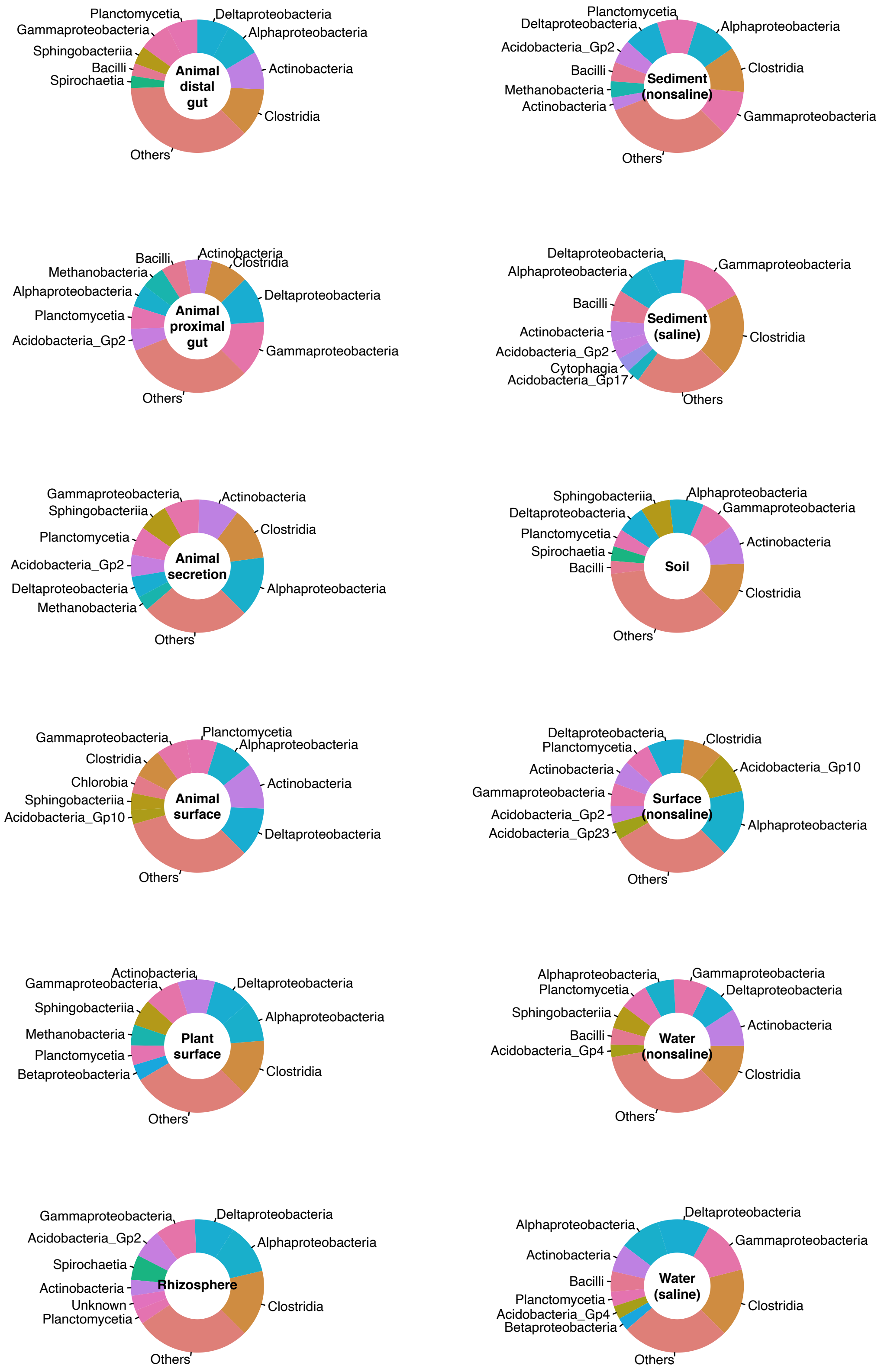

Figure S7. Taxon profiles of generalist edge linked vertices in subnetworks of 12 environments inferred from trimmed datasets

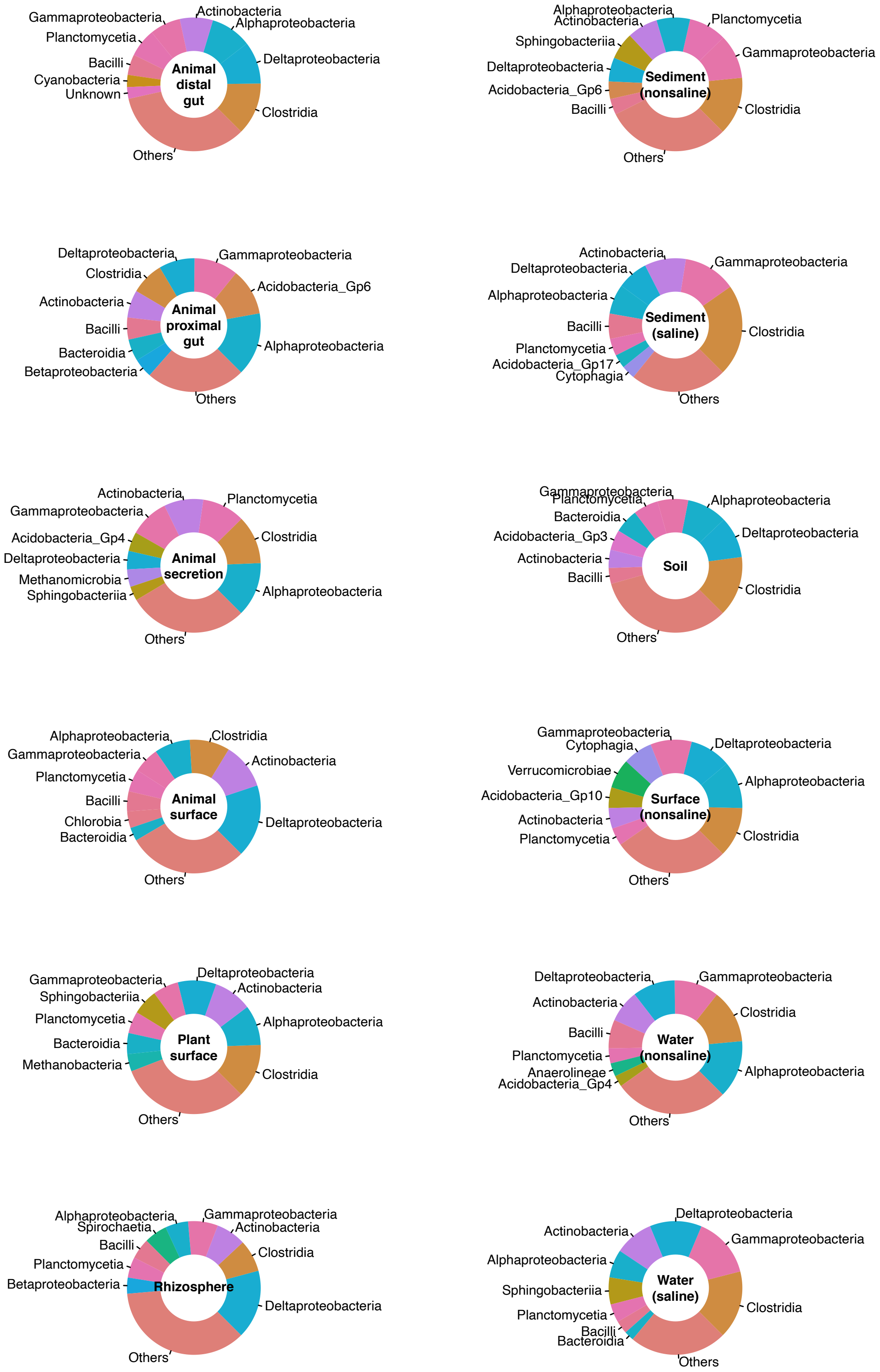

Figure S8. Taxon profiles of specialist edge linking generalist vertex pair linked vertices in subnetworks of 12 environments inferred from trimmed datasets

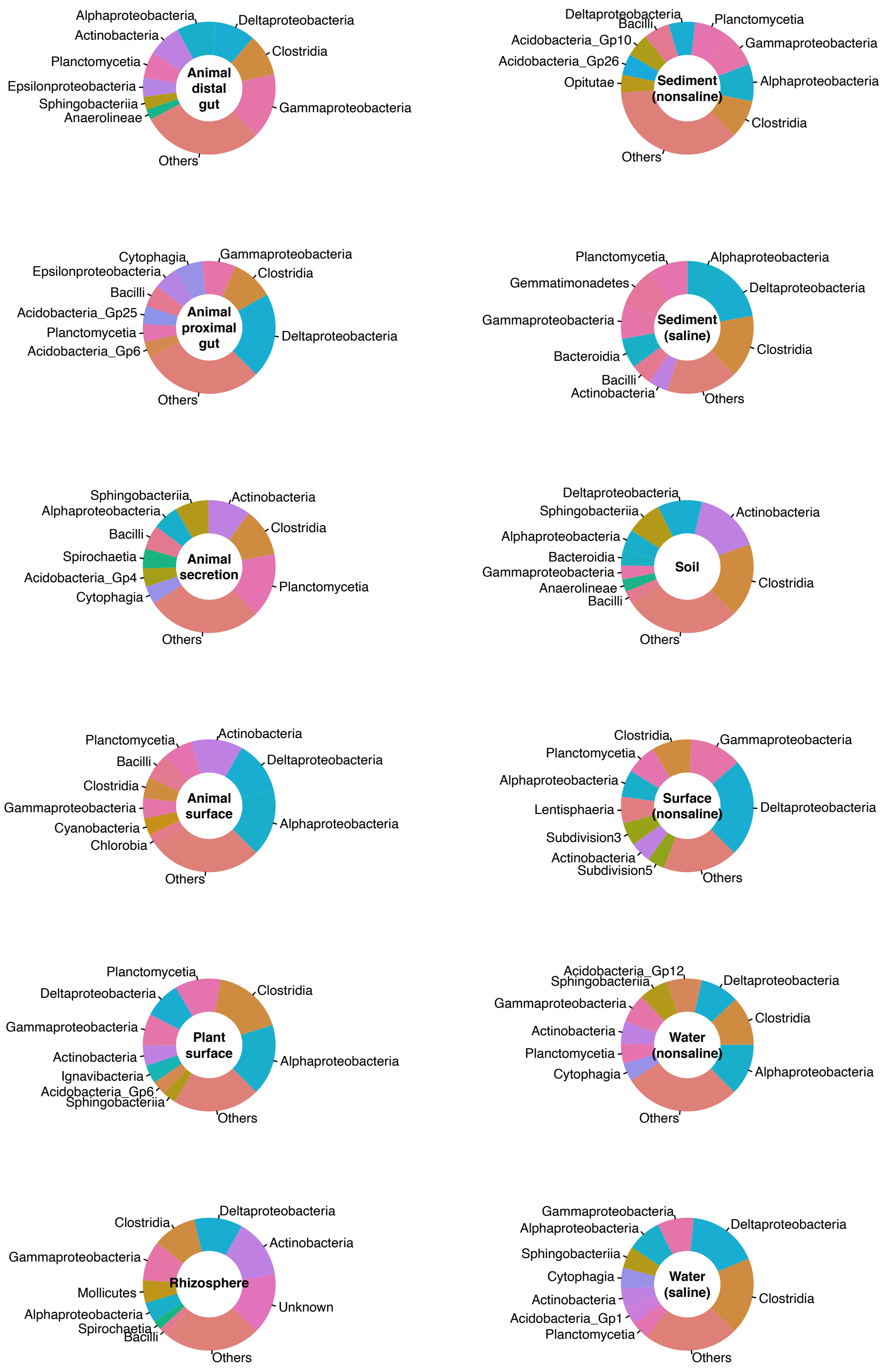

Figure S9. Taxon profiles of specialist edge linking specialist vertex pair linked vertices in subnetworks of 12 environments inferred from trimmed datasets

Table S1. The size of subnetworks and sample numbers for 14 environmental categories at level-3 of the EMP ontology.

Environment	EVS number	Edge number	Sample number
Water (nonsaline)	2820	15201	4914
Animal distal gut	2253	1179	4157
Soil	2092	2259	4278
Animal surface	1322	2241	2960
Sediment (nonsaline)	639	472	543
Plant surface	497	464	1610
Surface (nonsaline)	493	1209	1270
Water (saline)	481	1024	679
Sediment (saline)	462	122	558
Animal secretion	449	524	1256
Rhizosphere	422	966	553
Animal proximal gut	406	316	366
Plant corpus	209	141	124
Animal corpus	87	226	327
